# Differences in clinical features and gut microbiota between individuals with methamphetamine casual use and methamphetamine use disorder

**DOI:** 10.3389/fcimb.2023.1103919

**Published:** 2023-02-23

**Authors:** Li He, Bao-Zhu Yang, Yue-Jiao Ma, Li Wen, Feng Liu, Xiao-Jie Zhang, Tie-Qiao Liu

**Affiliations:** ^1^ Department of Psychiatry, and National Clinical Research Center for Mental Disorders, The Second Xiangya Hospital of Central South University, Changsha, Hunan, China; ^2^ Department of Psychiatry, Yale University School of Medicine, New Haven, CT, United States; ^3^ Department of Internal Medicine, Section of Endocrinology & Core Laboratory of Yale Center for Clinical Investigation, Yale University School of Medicine, New Haven, CT, United States; ^4^ Compulsory Detoxification Center of Changsha Public Security Bureau, Changsha, Hunan, China

**Keywords:** gut microbiome, clinical features, methamphetamine use disorder, casual use, network analysis

## Abstract

**Background:**

The transition from methamphetamine (MA) casual use (MCU) to compulsive use is enigmatic as some MA users can remain in casual use, but some cannot. There is a knowledge gap if gut microbiota (GM) play a role in differing MCU from MA use disorder (MUD). We aimed to investigate the clinical features and GM differences between individuals with MCU and MUD.

**Method:**

We recruited two groups of MA users –MCU and MUD – and matched them according to age and body mass index (n=21 in each group). Participants were accessed using the Semi-Structured Assessment for Drug Dependence and Alcoholism, and their fecal samples were undergone 16S ribosomal DNA sequencing. We compared the hosts’ clinical features and GM diversity, composition, and structure (represented by enterotypes) between the two groups. We have identified differential microbes between the two groups and performed network analyses connecting GM and the clinical traits.

**Result:**

Compared with the casual users, individuals with MUD had higher incidences of MA-induced neuropsychiatric symptoms (e.g., paranoia, depression) and withdrawal symptoms (e.g., fatigue, drowsiness, and increased appetite), as well as stronger cravings for and intentions to use MA, and increased MA tolerance. The GM diversity showed no significant differences between the two groups, but four genera (Halomonas, Clostridium, Devosia, and Dorea) were enriched in the individuals with MUD (p<0.05). Three distinct enterotypes were identified in all MA users, and Ruminococcus-driven enterotype 2 was dominant in individuals with MUD compared to the MCU (61.90% vs. 28.60%, p=0.03). Network analysis shows that Devosia is the hub genus (hub index = 0.75), which is not only related to the counts of the MUD diagnostic criteria (ρ=0.40; p=0.01) but also to the clinical features of MA users such as reduced social activities (ρ=0.54; p<0.01). Devosia is also associated with the increased intention to use MA (ρ=0.48; p<0.01), increased MA tolerance (ρ=0.38; p=0.01), craving for MA (ρ=0.37; p=0.01), and MA-induced withdrawal symptoms (p<0.05).

**Conclusion:**

Our findings suggest that Ruminococcus-driven enterotype 2 and the genera Devosia might be two influential factors that differentiate MA casual use from MUD, but further studies are warranted.

## 1 Introduction

Illicit drug use continues to be a significant public health concern worldwide. With an estimated 27 million users worldwide, amphetamine-type stimulants (ATSs) remain among the world’s most popular illicit drugs ((UNODC) TUNOoDaC, 2021). Methamphetamine (MA) is the most popular ATS, and its recreational use has increased over the past decade, particularly in East and Southeast Asia. In China, synthetic drug users (mainly MA) accounted for 55% of the nearly 2.2 million registered drug users in 2019, and the proportion of MA users has been increasing since the early 2000s ((UNODC) TUNOoDaC, 2021). The powerfully addictive nature of MA is one of the significant factors contributing to its misuse and addiction. The transition from casual use to habitual and compulsive use is commonly seen in individuals with MA use disorder (MUD). MA casual users can control drug-seeking and -taking behaviors, whereas individuals with MUD experience strong cravings for MA and lose self-control. Intriguingly, some MA casual users can stay casual use and never become MUD, while some MA casual users will transit to MUD. MA casual users and individuals with MUD have significant differences in their responses to MA; however, the differences in their clinical features are not entirely clear. Furthermore, the biological mechanism of MA casual use (MCU) to MUD transition has not yet been fully elucidated.

The human gut microbiota (GM) contains a genome of approximately 10 million genes, about 150-fold larger than the human genome (Meckel and Kiraly, 2019). With the emergence of the “gut-brain axis,” the influence of intestinal flora on the brain’s physiological, behavioral, and cognitive functions has become a trendy research topic (Wang and Wang, 2016). Recent studies have demonstrated a strong link between MA use and gut microbiome (Ning et al., 2017; Cook et al., 2019; Angoa-Pérez et al., 2020; Forouzan et al., 2020; Chen et al., 2021; Deng et al., 2021; Wang et al., 2021; Yang et al., 2021; Yang et al., 2021; Lai et al., 2022; Wang et al., 2022; Zhang et al., 2022). Clinical research has shown significant differences in the GM composition between MA users and non-MA users (Cook et al., 2019) and marked changes in GM among individuals with MUD related to their inflammatory markers and clinical characteristics (Deng et al., 2021; Yang et al., 2021). In animal studies, MA treatment disrupted the gut microbiome balance (Angoa-Pérez et al., 2020; Forouzan et al., 2020; Chen et al., 2021), and MA-induced conditioned place preference (CPP) resulted in dramatic changes in the diversity and composition of the GM (Ning et al., 2017; Wang et al., 2021; Yang et al., 2021). The GM continued to evolve during different phases of CPP, including acquisition, extinction, and reinstatement (Wang et al., 2021). These various lines of evidence suggest a potential link between GM and MUD development.

MA could disrupt the intestinal barrier and induce inflammatory responses, altering the gut microenvironment and potentially affecting GM structure (Persons et al., 2018; Wang et al., 2022). MA’s destruction of intestinal barrier integrity also leads to intestinal bacterial metabolites entering the circulation, potentially impacting the host (Wang et al., 2022; Zhang et al., 2022). It is also fascinating that GM consistently interacts with the host, and it is conceivable that the interactions between GM and the MA-using host are different in individuals with MCU and MUD. The altered microbiota structure might reflect the hosts’ MA use states, as clinical studies of individuals with MUD have suggested (Deng et al., 2021; Yang et al., 2021). Our previous animal study on MA use (Yang et al., 2021) suggested a link between GM and susceptibility to MA addiction. However, the differences in GM among individuals with different MA use statuses have rarely been studied. Building on previous findings, we aimed to investigate further the relationship between the GM, MCU, and MUD status, and specific clinical characteristics of MA users.

## 2 Methods

### 2.1 Study participants

We recruited the study participants from the Compulsory Detoxification Center of Changsha Public Security Bureau in Changsha, Hunan, China, between October 2018 and October 2019. All the study subjects had used MA within the previous 12 months. We recruited two groups of participants – one with MCU and the other with MUD. According to the Diagnostic and Statistical Manual of Mental Disorders, Fifth Edition (DSM‐5), MUD participants fulfilled at least 2 of 11 DSM-5 MUD diagnostic criteria, whereas MCU participants fulfilled 0 or 1 and could also control MA use. We assessed the participants using the Semi-Structured Assessment for Drug Dependence and Alcoholism (SSADDA), Chinese Version (Ma et al., 2021). The exclusion criteria of participants were as follows: 1) medical condition related to intestinal dysbacteriosis such as gastrointestinal disease, liver disease, or infection; 2) current neurological and psychiatric disorders not due to MA use; 3) antibiotics use in the previous three months; 4) other illicit substances used in the previous 12 months other than MA. The participants were given a complete explanation of the study before their invitation to participate. All participants signed written informed consent. The study was approved by the Ethics Committee of the Second Xiangya Hospital of Central South University.

Sixty-six participants (21 MCU and 45 MUD (42 males and 3 females)) were recruited for this study. None of the participants reported using antipsychotic medication or other prescription medications. We excluded the three female MUDs to avoid the confounding effect of sex and retained only the male participants in the subsequent investigation. To avoid the effects of age and obesity on GM, we conducted a propensity-matched analysis of the eligible participants by age and body mass index (BMI). To this end, 42 age- and BMI-matched participants (21 MCU and 21 MUD) were included in the current study.

### 2.2 Fecal sample collection and DNA preparation

The detoxification center provided the same meals for all the participants during their stay. After two weeks of detoxication, fecal samples from the participants were collected in sterile containers and immediately stored at -80°C until further processing. Bacterial DNA was extracted using the E.Z.N.A.^®^ Stool DNA Kit (Omega Bio-Tek, Norcross, GA, USA) according to the manufacturer’s protocols. The V4 regions of the bacteria 16S rRNA gene were amplified by a polymerase chain reaction (PCR) in triplicates with barcoded primers as previously described (Yang et al., 2021). PCR products were purified using the AxyPrep Mag PCR Clean-Up Kit (Axygen Biosciences, Union City, CA, USA), and the DNA concentration of each sample was assessed by Qubit 2.0 Fluorometer (Life Invitrogen, Carlsbad, CA, USA) according to the manufacturer’s guidelines.

### 2.3 16S rRNA gene sequencing and data processing

Purified PCR products were pooled to generate a library and sequenced on the Illumina MiSeq platform according to the standard protocols described by Genergy Biotechnology (Shanghai, China). Raw fastq files were demultiplexed, quality-filtered, and merged using the “DeBlur” algorithm in the QIIME2. Raw count data were filtered to remove low-expressed features with less than ten counts. Mitochondria and chloroplast were also filtered according to the taxonomy. Absolute Sequence Variants were generated using QIIME2 software program. The taxonomical assignment of absolute sequence variants was analyzed by QIIME2 against the GreenGenes database (gg_13_8) using a confidence threshold of 70%. The sequencing depth was unified to 20,000 counts for all samples to obtain relative abundance based on the alpha rarefaction curves (**Supplementary Figure 1**). The counts were converted to relative abundances by dividing total counts for further analysis. The raw sequencing data is available on the Sequence Read Archive (PRJNA910806).

### 2.4 Analysis of intestinal microbiota diversity

Alpha-diversity indexes were assessed using Wilcoxon rank sum tests, including Faith’s phylogenetic diversity, Shannon’s diversity, observed features, and Pielou’s evenness. The beta-diversity indexes, including Jaccard distance, Bray-Curtis distance, unweighted UniFrac distance, and weighted UniFrac distance, were calculated by performing a Permutational Multivariate Analysis of Variance (PERMANOVA).

### 2.5 Comparison of intestinal microbiota differences

Microbial differences between the MA casual and compulsive users were determined using Wilcoxon rank-sum tests and Linear discriminant analysis effect size (LEfSe). Linear discriminant analysis (LDA) values>2.0 at a *p*<0.05 were considered significantly enriched.

### 2.6 Analysis of enterotypes

An enterotype is a class of living microbes clustered according to their GM’s bacteriological composition (Arumugam et al., 2011). We analyzed enterotypes using the R packages ade4 (Chessel et al., 2004; Dray and Dufour, 2007; Dray et al., 2007; Bougeard and Dray, 2018; Thioulouse et al., 2018), cluster (Maechler et al., 2022), and clusterSim (Walesiak and Dudek, 2020). Enterotypes were identified based on the relative genus abundances using the Jensen-Shannon Distance (JSD) and the Partitioning Around Medoids (PAM) clustering algorithm and visualized by between-class analysis (BCA) (Arumugam et al., 2011).

### 2.7 Network analysis

Network analysis for the top 50 genera (accounting for approximately 99.9% of the abundance of all genera), enterotypes, and clinical traits was performed using the R package, psych (Revelle, 2022), and the p-values were adjusted by the false discovery rate. We defined an edge between two nodes to have statistically significant Spearman’s rank correlations (adjusted *p*< 0.05) with a magnitude of >= 0.35 or<= -0.35. We constructed undirected and directed network graphs to display the potential relationship between GM and MA-related clinical features. The network reconstruction and property measurements were conducted by Gephi 0.9.7 (Bastian. et al., 2009). The betweenness centrality index (BCI) is defined as


, $$\sum_{s\neq v\neq t\in V}\frac{\sigma_{st}\left(v\right)}{\sigma_{st}}$$

in which *V* is a set of nodes; *σ*_*st*_(*v*) denotes the number of shortest paths from node “s” to node “t” that a node v lies on, and **σ*_*st*_* indicates the number of shortest paths from *s* to *t* (Brandes, 2001). BCI was used to assess the importance of a node in the undirected network, and a node with a high BCI indicates its centrality in the network. The authority index and hub index are defined as


; $$x^{p}\leftarrow \sum_{q:\;\left(q,\;p\right)\in E}y^{q}$$

, $$y^{p}\leftarrow \sum_{q:\;\left(p,q\right)\in E}x^{q}$$

in which E represents a set of edges; *x* ^*p*^ denotes authority index by summing *y* ^*q*^ , for all *q* pointing to *p*. On the other hand, *y* ^*p*^ denotes hub index by summing *x* ^*q*^ for all *q* pointing to *p*) (Kleinberg, 1999). These two indexes were used to compare the magnitude of in- and out-degree in the directed network. In the directed network, GM having higher hub indexes is more important in relation to MA-induced clinical features, while clinical features having higher authority indexes were more critical in association with GM. The Fruchterman and Reingold algorithms are used in Gephi to create the graph layout (Thomas Fruchterman, 1991).

### 2.8 Statistical analyses

Statistical analyses were performed using SPSS 26.0. For demographic and clinical characteristics, differences between the two groups were assessed using the chi-square tests for categorical variables, Wilcoxon rank-sum tests for continuous variables, and the *Z*-test for comparison of percentages. When sample sizes were small (n<=5), the Fisher exact test was employed instead of the chi-square test. Continuous variables were represented by the median (interquartile range), and categorical variables were described as the number. *P*<0.05 was considered statistically significant.

## 3 Results

### 3.1 Characteristics of participants

**Table 1** shows the baseline information for the MCU and MUD participants. There were no significant differences between the two groups in age, age of the first MA use, BMI, education levels, or life status, including adoptee, employment, marriage, or having children. However, compared to the MCU group, the MUD group had significantly more MA withdrawal episodes (median counts: 1 vs. 0, *p<*0.01) and longer durations of overall MA use (median years: 2 vs. 0, *p<*0.01), of daily MA use (median months: 3 vs. 0, *p<*0.01), and of heaviest use (median months: 4 vs. 1, *p<*0.01). The MUD group also spent more time (median days: 30 vs. 3, *p<*0.01) and money (median yuan: 300 vs. 100, *p<*0.01) on MA during the heaviest use periods, and their withdrawal symptoms lasted longer (median days: 3 vs. 0, *p<*0.01).

**Table 1:** Demographic and clinical characteristics of the study participants: Group comparison between methamphetamine (MA) casual use and MA use disorder.

Characteristics	MA casual use (n=21)	MA use disorder (n=21)	*p*-value
Age, median (IQR)	37 (33, 41)	36 (29, 44)	0.70
Body mass index (kg/m^2^), median (IQR)	24.22 (21.1, 24.9)	24.06 (21.2, 25.7)	0.73
Year of education, median (IQR)	9 (8, 12)	9 (9, 12)	0.41
Adoption, n (%) No	20 (95.2)	20 (95.2)	1
Yes	1 (4.8)	1 (4.8)	
Employment, n (%) Have jobs	8 (38.1)	9 (42.9)	0.75
Have no job	13 (61.9)	12 (57.1)	
Marriage, n (%) Married	9 (42.9)	7 (33.3)	0.52
Widowed	0 (0)	1 (4.8)	
Divorced	7 (33.3)	5 (23.8)	
Never married	5 (23.8)	8 (38.1)	
Children, n (%) 0	5 (23.8)	6 (30.0)	0.74
1	12 (57.1)	9 (45.0)	
≥2	4 (19.0)	5 (25.0)	
Counts of MUD criteria, median (IQR)	0 (0, 0)	7 (4, 10)	0.00^*^
Age of first MA use, median (IQR)	30 (27, 36)	31 (22, 37)	0.44
Year of MA use, median (IQR)^†^	0 (0, 0)	2 (0, 3)	0.00^*^
MA withdrawal episodes, median (IQR)^‡^	0 (0, 0)	1 (0, 1)	0.00^*^
Maximum duration of daily MA use (month), median (IQR)	0 (0, 0)	3 (0, 12)	0.00^*^
Duration of heaviest use (month), median (IQR)	1 (1, 2)	4 (2, 12)	0.00^*^
Days of MA use per month, median (IQR)^#^	3 (2, 5)	30 (8, 30)	0.00^*^
Daily cost for MA (yuan), median (IQR)^#^	100 (100, 200)	300 (200, 350)	0.00^*^
Number of times MA withdrawal symptoms occur together, median (IQR)	0 (0, 0)	0 (0, 11)	0.13
Maximum duration of the withdrawal symptoms occurred together (day), median (IQR)	0 (0, 0)	3 (1, 6)	0.00^*^

^*^p<0.01; ^†^include only those years in which the subject used MA at least three times/week for a month or more for at least six months; ^#^during the heaviest use; IQR, interquartile range; ^‡^did not use MA for three months or longer. n, count.

To further compare the differences in clinical features between the two groups, we summarized the relevant yes/no answers from the MA section of the SSADDA Chinese version, including 19 facets and 74 questions (**Supplementary Table 1**). In addition to the MUD group that had more frequent use of MA and more cravings, they uniquely exhibited a loss of control over MA use, as evidenced by a higher incidence of inability to stop MA use (52.4% vs. 0.0%, *p<*0.01). The individuals in the MUD group also had a greater intention to use MA (57.1% vs. 0.0%, *p<*0.01) and higher tolerance to MA (47.6% vs. 0.0%, *p<*0.01). Moreover, the MUD group showed higher symptom rates of paranoia (23.8% vs. 0.0%, *p<*0.05) and depression (23.8% vs. 0.0%, *p<*0.05) during MA use, while they experienced a higher incidence of fatigue (81.0% vs. 14.3%, *p<*0.01), drowsiness (81.0% vs. 14.3%, *p<*0.01), and increased appetite (66.7% vs. 4.8%, *p<*0.01) during withdrawal. In further contrast to MCU, MUD participants were more likely to engage in risky behaviors under the influence of MA (57.1% vs. 9.5%, *p<*0.01) and demonstrated neglecting major work of job, household, and family responsibilities (57.1% vs. 0.0%, *p<*0.01). Furthermore, individuals in the MUD group showed a reduction in essential activities (57.1% vs. 0.0%, p<0.01), which was accompanied by significantly greater damage to their social lives, such as reducing contact with friends or family (57.1% vs. 4.8%, *p*<0.01) and having problems with friends or family (85.7% vs. 52.4%, p=0.02) because of MA use.

### 3.2 Composition and diversity of the microbial community

**Figure 1:**
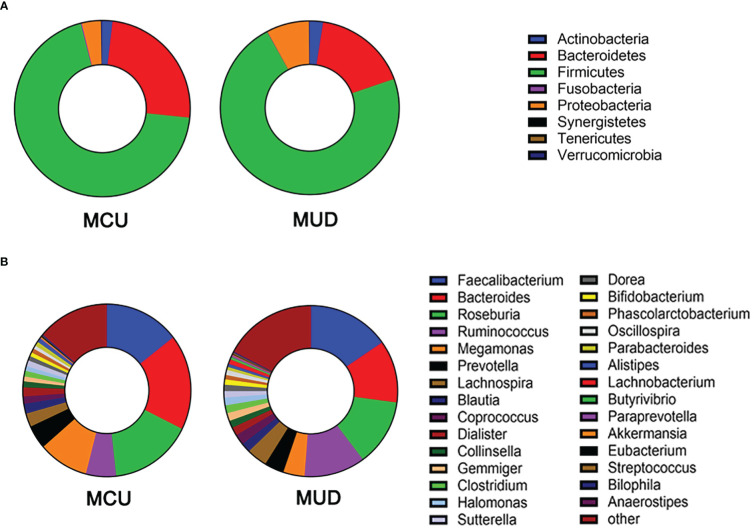
Gut microbiota composition of methamphetamine (MA) users at phylum and genus level. **(A)** The average bacterial compositions at the phylum level. **(B)** The top 30 genera in terms of average relative abundance. MCU, individuals with MA casual use; MUD, individuals with MA use disorder.

After size filtering, quality control, chimera removal, and rarefying, a total of 840,000 high-quality sequence reads were acquired from the fecal samples of 42 participants. These sequences were clustered into 1,721 absolute sequence variants, followed by assigning them to 8 phyla, 15 classes, 23 orders, 49 families, and 94 genera. **Figure 1** shows the average bacterial compositions of MCU and MUD participants at the phylum and genus levels. Firmicutes (average relative abundance 69.4% vs. 72.5%), Bacteroidetes (24.8% vs. 17.2%), Proteobacteria (3.5% vs. 7.7%), and Actinobacteria (1.9% vs. 2.4%) were the four dominant phyla for the MCU and MUD groups, respectively, accounting for more than 90% of the intestinal flora. Faecalibacterium (average relative abundance 14.5% vs. 15.4%), Bacteroides (17.9% vs. 11.8%), Roseburia (15.9% vs. 12.6%), Ruminococcus (5.8% vs. 11.4%), Megamonas (9.4% vs. 4.0%), Prevotella (4.5% vs. 3.7%), Lachnospira (2.6% vs. 3.8%), Blautia (1.8% vs. 1.8%), Coprococcus (1.4% vs. 2.0%), and Dialister (1.7% vs. 1.6%) were the top ten genera among the MCU and MUD groups, respectively. However, we did not find significant differences in the GM’s alpha and beta diversity between MCU and MUD groups (**Table 2**).

**Table 2:** Differences in the diversity of microbial communities among the participants using methamphetamine (MA): Group comparison between MA casual use and MA use disorder.

Indexes		MA casual use	MA use disorder	*p*-value
Alpha diversity, median (IQR)	Faith’s phylogenetic diversity	9.20 (8.16, 10.22)	9.34 (7.87, 11.19)	0.59
	Shannon’s diversity	4.67 (4.35, 4.92)	4.95 (4.46, 5.21)	0.13
	Pielou’s evenness	0.61 (0.55, 0.64)	0.64 (0.60, 0.66)	0.16
	Observed features	219 (162, 234)	215 (176, 245)	0.53
Beta diversity, median (IQR)	Jaccard distance^a^	0.92 (0.86, 0.98)	0.91 (0.86, 0.98)	0.84
	Bray-Curtis distance^a^	0.99 (0.96, 1.00)	0.99 (0.96, 1.00)	0.68
	Weighted unifrac distance^a^	0.32 (0.25, 0.39)	0.30 (0.23, 0.38)	0.07
	Unweighted unifrac distance^a^	0.41 (0.37, 0.45)	0.39 (0.36, 0.45)	0.97

IQR, inter-quartile range; ^a^, distances to MA casual use.

### 3.3 Analysis of intestinal microbiota differences between the MCU and MUD groups

**Figure 2:**
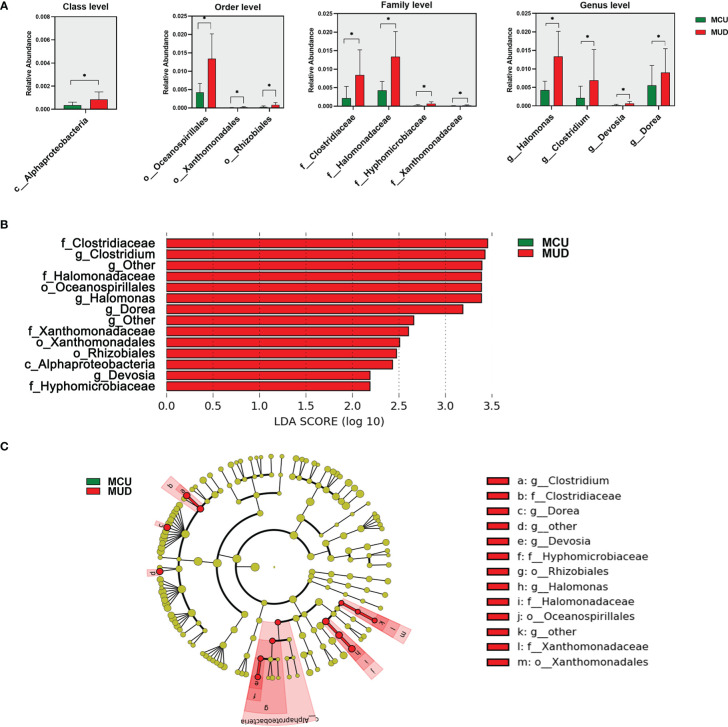
Differences in gut microbiota between individuals with methamphetamine (MA) casual use and MA use disorder. **(A)** The taxa with significant differences (*p*<0.05) between MCU and MUD were determined by the Wilcoxon rank-sum test. **(B)** The taxa significant differences (LDA score>2.0 and *p*<0.05) between MCU and MUD were detected by the LEfSe analysis. **(C)** The cladogram shows the differential taxa between the MCU and MUD found in the LEfSe analysis. *p<0.05; MCU, individuals with MA casual use; MUD, individuals with MA use disorder; the green color represents the MCU group; the red color represents the MUD group.

We used Wilcoxon rank-sum tests to identify differential taxa between the MCU and MUD groups (**Figure 2A**). The analysis revealed a significant increase in the relative abundance of some taxa in the MUD group. At the class level, the relative abundance of Alphaproteobacteria was significantly higher in the MUD group compared with the MCU group (*p*=0.03). At the order level, Oceanospirillales, Xanthomonadales, and Rhizobiales were significantly higher in the MUD group than in the MCU group (*p*=0.02, *p*=0.02, and *p*=0.03, respectively). Further, four families, Clostridiaceae, Halomonadaceae, Hyphomicrobiaceae, and Xanthomonadaceae, were significantly higher in the MUD group than in the MCU group (*p*=0.01, *p*=0.02, *p*=0.02, and *p*=0.02, respectively). The genera of Halomonas, Clostridium, Devosia, and Dorea were significantly higher in the MUD group than in the MCU group (*p*=0.02, *p*=0.02, *p*=0.02, and *p*=0.04, respectively). We further confirmed these results using the LEfSe analysis (**Figures 2B, C**), consistent with those using Wilcoxon rank-sum tests. For validation, we compared the 21 MCUs to the 21 unmatched MUDs (those excluded by the propensity-matched analysis) using the LEfSe analysis. Still, these two groups differed significantly in age (median age 37 vs. 29, *p<* 0.01). The genus Clostridium remained significantly more abundant in the unmatched MUD group than in the MCU group (LD score = 3.00, *p*= 0.03). In other words, we replicated one of the four genera showing the difference in abundance between the MCU and MUD groups. However, we could not exclude the confounding effects of age in this validation analysis. An in-depth elaboration is presented in the discussion.

### 3.4 Analysis of enterotypes

**Figure 3:**
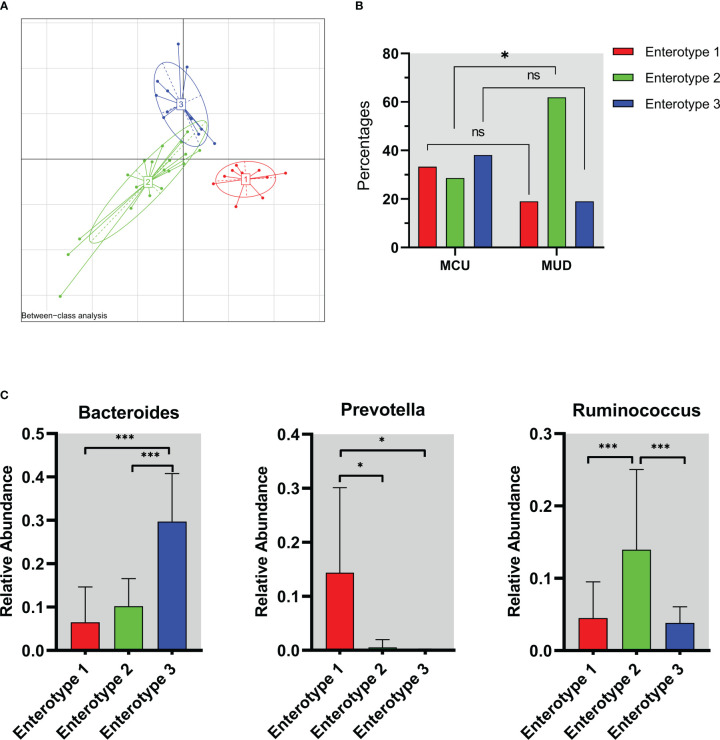
The enterotype characteristics of methamphetamine (MA) users. **(A)** The result of Between-class analysis. The genus taxa clustered into three groups based on the Jensen-Shannon divergence among the MA users. **(B)** Distribution of enterotypes in MCU and MUD. The percentage of enterotype 2 was higher in the MUD compared to the MCU (61.9% vs. 28.6%, p=0.03). **(C)** The abundance of representative genera in different enterotypes. Prevotella (*p*=0.01), Ruminococcus (*p*<0.01), and Bacteroides (*p*<0.01) were enriched in enterotype 1, enterotype 2, and enterotype 3, respectively. *p<0.05; **p<0.01; MCU, individuals with MA casual use; MUD, individuals with MA use disorder; the red color represents enterotype 1; the green color represents enterotype 2; the blue color represents enterotype 3. ns, no significance.

We assessed the enterotypes using Between-class analysis (BCA). The results showed that the genus taxa clustered into three groups based on the Jensen-Shannon divergence (JSD) among the MA users (**Figure 3A**), suggesting three different enterotypes were present. The percentage of enterotype 2 was higher in the MUD group compared to the MCU group (61.9% vs. 28.6%, *p*=0.03), while enterotype 1 (19.0% vs. 33.3%, *p*=0.27) and enterotype 3 (19.0% vs. 38.1%, *p*=0.16) were not significantly different between the two groups (**Figure 3B**). To further understand the features of enterotypes identified in all of the MA participants, we analyzed the abundances of three representative genera (Bacteroides, Prevotella, and Ruminococcus) among these different enterotypes (Arumugam et al., 2011). Prevotella (*p*=0.01), Ruminococcus (*p<*0.01), and Bacteroides (*p<*0.01) were enriched in enterotype 1, enterotype 2, and enterotype 3, respectively (**Figure 3C**).

### 3.5 Network analysis

**Figure 4:**
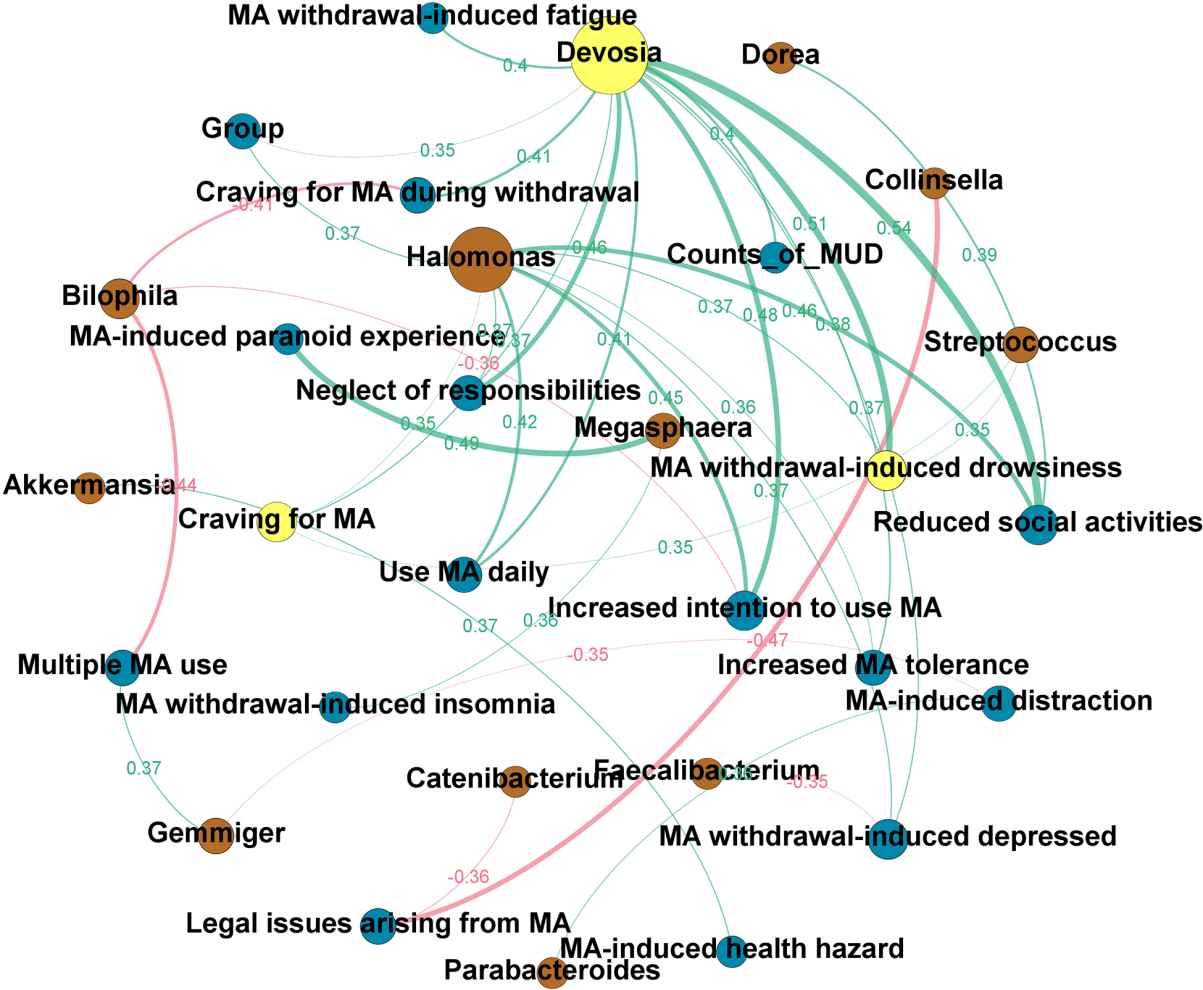
The clinical-microbial network in methamphetamine (MA) users. A directed network from the microbiota to clinical traits of MA users was built. The node size reflected the number of ‘degree,’ in which ‘degree’ is defined as the number of edges directly connected to nodes in the network. Yellow node indicates the hub genus or clinical trait in the clinical-microbial network. Brown node indicates the intestinal flora at the genus level. Blue node indicates the clinical traits of MA users (c.f. **Supplementary Table 1** for the details). The green line indicates a positive correlation, while the red line indicates a negative correlation. The width of the line represents the magnitude of the absolute value of Spearman’s correlation coefficient between the two nodes; the broader the width, the greater the correlation coefficient. The exact Spearman’s correlation coefficient values are also shown in the graph, in the same color as the line to which they belong.

**Figure 4** shows the directed network. The node size reflected the number of ‘degree,’ in which ‘degree’ is defined as the number of edges directly connected to the nodes in the network. Based on the hub index, Devosia was the hub genus, which was related to many of the clinical features we identified to be significantly increased in the MUD (hub index = 0.75). These clinical features include reduced social activities (*ρ*=0.54; *p*<0.01), increased intention to use MA (*ρ*=0.48; *p*<0.01), neglect of responsibilities (*ρ*=0.46; *p*<0.01), MA use daily (*ρ*=0.41; *p*=0.01), increased MA tolerance (*ρ*=0.38; *p*=0.01) and craving for MA (*ρ*=0.37; *p*=0.01), as well as MA-induced withdrawal symptoms, such as drowsiness (*ρ*=0.51; *p*<0.01), craving during withdrawal (*ρ*=0.41; *p*=0.01), fatigue (*ρ*=0.40; *p*=0.01), and depressed (*ρ*=0.37; *p*=0.02). Besides, the genus Devosia was also related to the counts of MUD diagnostic criteria (*ρ*=0.40; *p*=0.01) and MA use status, i.e., MCU or MUD (*ρ*=0.35, *p*=0.02). The clinical features most associated with GM are craving for MA and MA withdrawal-induced drowsiness, all of which yield an authority index of 0.34. To explore the relationships of specific GM to GM, clinical traits to clinical traits, and GM to clinical traits, we also constructed an undirected network (see **Supplementary Figure 2**). We found three hub nodes in this network, i.e., enterotype, genus Devosia, and genus Halomonas, and the genus Devosia was highly associated with the genus Halomonas (*ρ*=0.91, *p*<0.01). Besides, in the undirected network, the enterotype only connected to microbiota nodes, particularly two nodes, Bacteroides (*ρ*=0.7, *p*<0.01) and Prevotella (*ρ*=-0.38, *p*=0.01), which were enriched in enterotype 3 and enterotype 1, respectively.

## 4 Discussion

There is a significant distinction in response to MA between MA casual users and individuals with MUD. Our study found substantial differences in clinical features between the two groups. In addition to more significant cravings, higher intention to use MA, and increased MA tolerance, MUD patients had higher incidences of MA-induced neuropsychiatric symptoms, withdrawal symptoms, social damage, and neglecting responsibilities. Notably, MCU participants showed better self-control and the ability to limit MA use than individuals with MUD. Besides, we also found differences in the composition of GM, including specific microbes in various classes, orders, and families, between the two groups of MA use. Our study also suggests a link between GM and the clinical features of MA users and indicates a potential role of GM in whether a transition occurs from MCU to MUD.

MA users were found to have relatively longer transitions from the onset of drug use to compulsive use than heroin users (85.0 vs. 50.0 days) (Wang et al., 2017). Although the core factors that cause the transition from MCU to MUD are unclear, several lines of evidence suggest that the dose of MA, frequency of use, and biological factors influence the response to MA (Zhang et al., 2020; McKetin et al., 2013; Rau et al., 2016; Mayo et al., 2019).. Our study showed that MUD patients spent more time and money on MA and had more prolonged MA use than MCU participants, supporting earlier findings that repetitive drug use could develop MUD (Tien and Ho, 2011; Mizoguchi and Yamada, 2019). Moreover, our study found that individuals with MUD had more obvious MA-induced neuropsychiatric and withdrawal symptoms than the MCU group. MA-dependent users had a high incidence of substance-induced psychotic disorders (23.8%), particularly delusions (16.4%) and hallucinations (14.8%) (Salo et al., 2011), and MA-related psychiatric symptoms were associated with the MA dose and duration of MA use (McKetin, 2018). Withdrawal symptoms are closely associated with MA relapse and low treatment compliance (Zorick et al., 2010; Pennay and Lee, 2011). Depression and psychotic symptoms were most prevalent among MA-dependent individuals during MA abstinence (Zorick et al., 2010). MA withdrawal symptoms were also associated with more frequent MA use (*p*<0.05) (Zhao et al., 2021). Recent studies have shown that biological factors, such as genetics or neuroimmunology, are associated with MA addiction (Zhang et al., 2020; Mayo et al., 2019; Shi et al., 2022). However, the field of treating MUD still as yet lacks biological intervention targets (Shoptaw et al., 2009; Morley et al., 2017). Given the increased intensity of MUD patients’ MA cravings, withdrawal symptoms, and ensuring knock-on effects on treatment efficacy, investigating the differences between MCU and MUD participants is a crucial step to elucidate and thus target the biological mechanisms of MUD’s development.

Gut flora is a promising target for addiction treatment, as clinical studies have shown that antibiotics, probiotics, and fecal transplants effectively reduce alcohol-induced somatic symptoms and cravings (Kirpich et al., 2008; Zuo et al., 2017; Bajaj et al., 2021). In addition, a growing number of studies have found that using non-intestinally absorbed antibiotics could affect the development of addiction in various animal models (Kiraly et al., 2016; Chen et al., 2020; Ezquer et al., 2021). Recent studies have demonstrated that the composition of intestinal microflora was altered in patients with alcohol, opioids, cocaine, or MA use disorders (Mutlu et al., 2012; Volpe et al., 2014; Acharya et al., 2017; Cook et al., 2019). Moreover, these changes were related to the behavioral changes of experimental animals in substance-induced animal models (Ning et al., 2017; Scorza et al., 2019). However, GM’s role in developing substance use disorders remains unclear. To clarify the association between GM and the development of MUD, we conducted a comparative analysis of intestinal microflora between individuals with MCU and MUD. We found that four genera (Halomonas, Clostridium, Devosia, and Dorea) dramatically increased with MUD and stated their appealing relevance to our study as follows:

The species of the genus Halomonas are Gram-negative aerobic bacteria with salt tolerance (Kim et al., 2013; Gasperotti et al., 2018). Genus Halomonas with pathogenic potential could cause bacteremia, particularly in a dialysis setting (Kim et al., 2010; Stevens et al., 2013). To our knowledge, we are the first to report the link between Halomonas and substance use disorder. Previously, Halomonas was found to be significantly elevated in the intestines of HIV-infected patients and was associated with sexual transmission of HIV (Xu et al., 2021).

Species of the genus Clostridium are Gram-positive and anaerobic bacteria that could produce short-chain fatty acids (SCFAs) (Dürre, 2014). Of note, SCFAs are often beneficial to health. As another layer of complexity differs in the impact, some strains of the genus Clostridium produce short-chain fatty acids and are considered probiotics (e.g., Clostridium butyricum) (Chen et al., 2020). In contrast, some strains are considered pathogenic (e.g., Clostridium difficile) (Kuehne et al., 2010). Thus, the effects of the genus Clostridium are not exclusively related to SCFA. Our study found an association between Clostridium and MUD with higher abundance in the MUD than in the MCU group. Other studies (Peterson et al., 2017; Fulcher et al., 2018) have found Clostridium also to be related to alcohol and marijuana use, which suggested a role in substance use. Note that the genus Clostridium expression remained significantly higher in the MUD group than in the MCU group for the validation analysis using the unmatched MUDs. We further analyzed the demographic traits of the two MUD groups (i.e., age-matched and -unmatched). We found no differences between these two groups in BMI (*p* = 0.59), years of MA use (*p* = 0.58), MA withdrawal episodes (*p* = 0.50), and counts of DSM-5 MUD diagnostic criteria (*p* = 0.09), except for significant differences in age (*p*< 0.01) and age of first MA use (*p*< 0.01). The above new evidence suggests that the relationship between the genus Clostridium and MUD may be independent of the age and age of first MA use. However, we cannot exclude the confounding effects of age and age of first MA use in the genera Devosia, Dorea, and Halomonas between MCU and unmatched MUD group. Intriguingly, Clostridium was reported to play a role in metabolizing MA to amphetamine (Caldwell et al., 1972; Caldwell and Hawksworth, 1973). In sum, our findings suggest Clostridium differs MUD from MCU and might trigger the transition from MCU to MUD.

Species of the genus Devosia are Gram-negative and aerobic bacteria (Yoon et al., 2007). A recent study reported a significant increase in Devosia in patients with colorectal cancer, which might be a promising biomarker for the early detection of colorectal cancer (Zhang et al., 2019). Our study revealed a significant increase in Devosia in individuals with MUD, and the abundance of Devosia was correlated to the counts of the DSM-5 MUD diagnostic criteria. Furthermore, the analysis of the clinical-microbial network shows that Devosia is the hub genus and is associated with the clinical features that we identified to be significantly increased in the MUD.

Another associated gut microbe we identified is Dorea. Previously, Dorea was positively correlated with obesity (Zeng et al., 2019; Pinart and Dötsch, 2021). Dorea also increased significantly in patients with irritable bowel syndrome (Maharshak et al., 2018; Liu et al., 2021). One study has shown a significantly lower level of Dorea in patients with MUD compared with a healthy control (Deng et al., 2021), whereas our study showed an increase of Dorea in individuals with MUD compared with MA casual users. These indicated that Dorea might play a more complex role in various MA use statuses.

In addition, our network analysis showed that two of the clinical features of MA users, craving for MA and MA withdrawal-induced drowsiness, were significantly associated with the altered gut flora, suggesting a potential role of gut flora in the development of MA addiction. Our previous animal studies (Yang et al., 2021) showed a relationship between gut flora and the MA-induced CPP scores (i.e., measurements of animals’ MA liking). The altered gut flora by non-intestinal absorbable antibiotics could affect the CPP scores, suggesting an effect of gut flora on susceptibility to MA addiction.

Enterotypes are clusters of gut microbial communities that share similar bacteria compositions, which are not always stable and can be affected by diet, age, and antibiotics (Arumugam et al., 2011; Cheng and Ning, 2019). Research has found associations between enterotypes and opioid agonists (Gicquelais et al., 2020). Our study identified three distinct enterotypes (Bacteroides, Prevotella, and Ruminococcus) in MA users. Enterotype 2, in which Ruminococcus dominates, was present in more than half of our participants with MUD. The Ruminococcus-driven enterotype was not widespread and might not exist in some populations, in contrast to the Bacteroides-driven and Prevotella-driven enterotypes (Arumugam et al., 2011). A Han Chinese and Muslim (Li et al., 2018) study found only two different enterotypes (Bacteroides and Prevotella) (Fulcher et al., 2018). In a Taiwanese cohort, the Ruminococcus-driven enterotype was absent, while the Bacteroides- and Prevotella-driven enterotypes were present (Liang et al., 2017). Ruminococcus-driven enterotype predominates in certain diseases, such as Parkinson’s disease and obstructive sleep apnea-hypopnea syndrome (Ko et al., 2019; Zhang et al., 2022). Moreover, the enterotypes are influenced by the intestinal flora’s richness and diversity and are related to the absorption and metabolism of substances (Liang et al., 2017; Vandeputte et al., 2017; Brial et al., 2021). Several studies have shown that the Bacteroides-driven enterotype is related to a protein and animal fat diet, while the Prevotella-driven enterotype is associated with a carbohydrate diet (Wu et al., 2011). Our network analysis suggests that gut flora, such as Prevotella and Bacteroides, are associated with enterotypes, although we did not find any MA users’ clinical features directly correlated with enterotypes. Further study is needed to determine the defined effects of these enterotypes on the hosts.

The limitations of the current study are as follows. First, all the study participants are men, so we cannot generalize the influence of intestinal flora on MUD to the different sex. As noted earlier, the sample size of this study is limited. Ideally, multicenter studies and extensive sample recruitment will be needed to prove the role of intestinal microflora changes in developing MUD. To exclude the effects of diet, age, and obesity on the intestinal flora, in our current study, we collected stool samples from age- and BMI- matched participants after two weeks of a common diet source in a compulsory drug rehabilitation center. Also, the detoxification treatment (i.e., no MA intake while the participants were residing in the detoxification center) may have altered some extent of the MA’s effects on intestinal flora (Forouzan et al., 2020; Wang et al., 2021).

In summary, our study investigated the differences in clinical characteristics and GM between individuals with MCU and MUD. We found that they differed significantly in drug use patterns and MA-induced symptoms. We showed that the composition of GM significantly differed in individuals with MUD, and these differences were associated with the clinical characteristics of MA users. Our study may suggest a potential link between the microbiota and the progression from MCU to developing MUD.

## Data availability statement

The data presented in the study are deposited in the Sequence Read Archive, accession number PRJNA910806.

## Ethics statement

The studies involving human participants were reviewed and approved by Ethics Committee of the Second Xiangya Hospital of Central South University. The patients/participants provided their written informed consent to participate in this study.

## Author contributions

LH conducted the data analysis and drafted and revised the manuscript. B-ZY guided the methodology and interpreted the result; she also wrote and revised the manuscript. Y-JM recruited the study participants and curated the data. LW commented on and revised the manuscript. FL provided the resources during the participant recruitment. X-JZ designed and supervised the study. T-QL is the principal investigator for the study, supervising and providing resources for the current study. All authors contributed to the article and approved the submitted version.
