# Supplementary figures and images for: Differences in clinical features and gut microbiota between individuals with methamphetamine casual use and methamphetamine use disorder
Source: Front Cell Infect Microbiol. 2023 Feb 23;13:1103919. doi: 10.3389/fcimb.2023.1103919 (PMC9996337; doi:10.3389/fcimb.2023.1103919)

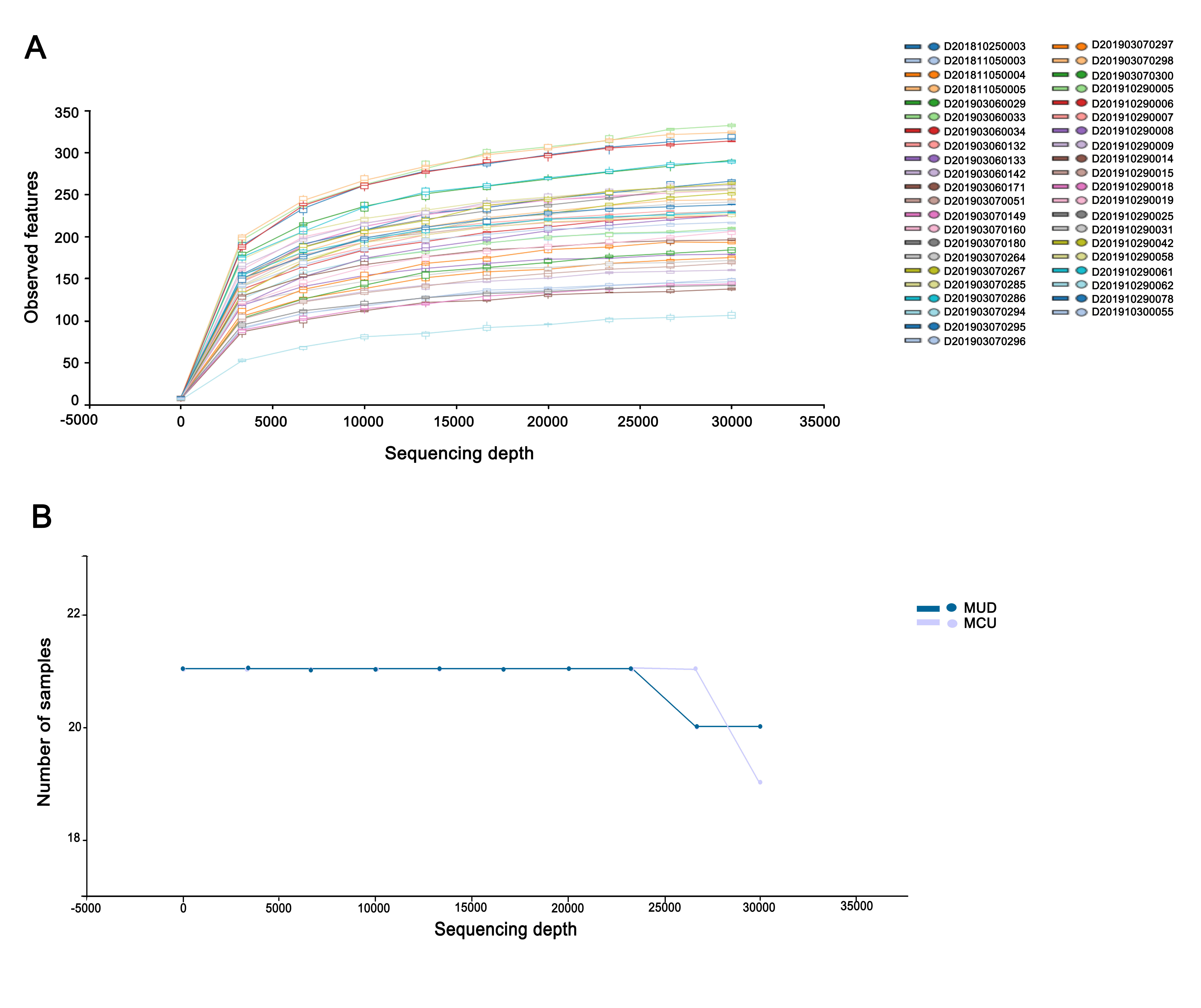

Supplement: Supplementary file 1 [file Image_1.jpeg]

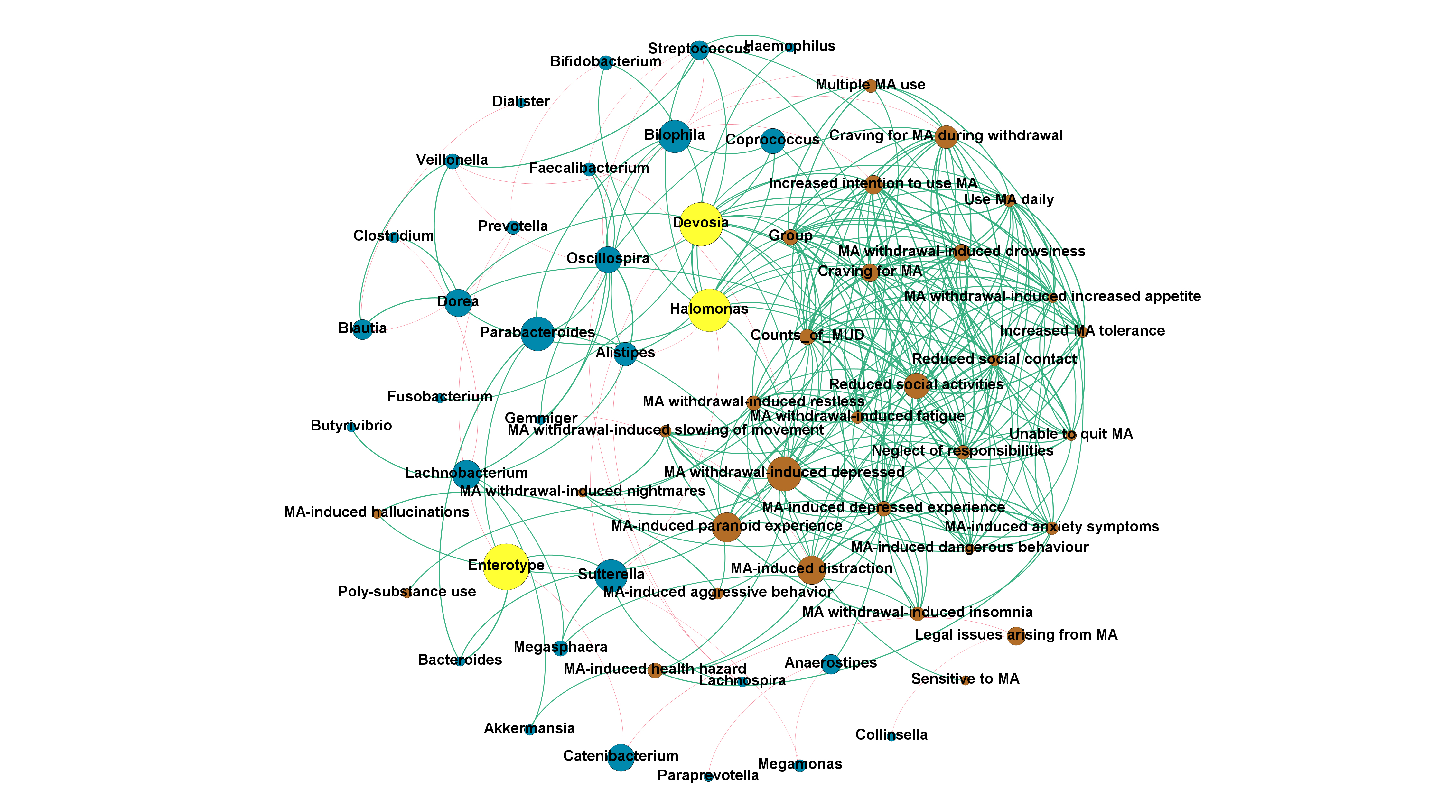

Supplement: Supplementary file 2 [file Image_2.jpeg]
