# Supplementary material for: Differences in clinical features and gut microbiota between individuals with methamphetamine casual use and methamphetamine use disorder
Source: Front Cell Infect Microbiol. 2023 Feb 23;13:1103919. doi: 10.3389/fcimb.2023.1103919 (PMC9996337; doi:10.3389/fcimb.2023.1103919)
Supplement: Supplementary file 3 [file Table_1.docx]

Supplementary table1 Questionnaire on methamphetamine use based on the Semi-Structured Assessment For Drug Dependence And Alcoholism (SSADDA)

| Facets | Question codes | Contents | | MA casual users | MA addicts | p-value |
| --- | --- | --- | --- | --- | --- | --- |
| / | F1 | Have you ever used methamphetamine？(yes/no) | | 21/0 | 21/0 | / |
| 1. Frequency of use | F1A1 | Would you say 11 or more times in your life have you used methamphetamine? (yes/no) | | 11/10 | 16/5 | 0.12 |
|  | F1C1 | Did you use methamphetamine at least 11 times during the past 12 months? (yes/no) | | 2/19 | 12/9 | <0.01 |
|  | F1D | Did you ever use methamphetamine at least once a week for a month or more? (yes/no) | | 6/15 | 17/4 | <0.01 |
| 2. Use during adolescence | F2A | Did you use methamphetamine more than once before you were 15? (yes/no) | | 0/21 | 0/21 | / |
| 3. sensitive | F3F | When you first started using methamphetamine, did you find that you got higher or stayed higher longer than other people who would use the same amount of methamphetamine? (yes/no) | | 5/16 | 4/17 | 0.71 |
| 4. Combined use of other substances | F3G | Did you ever use alcohol or any other drug to make yourself feel better when coming down from the effects of methamphetamine? (yes/no) | | 2/19 | 6/15 | 0.12 |
|  | F19 | Have you ever used methamphetamine together with one or more other drugs, including alcohol? (yes/no) | | 3/18 | 7/14 | 0.15 |
| 5. Time spend using | F3 | Did you ever use methamphetamine daily or almost daily? (yes/no) | | 0/21 | 14/7 | <0.01 |
|  | F4 | Have you ever stayed high from methamphetamine for a whole day or more? (yes/no) | | 2/19 | 15/6 | <0.01 |
|  | F4A | IF YES: Did this happen 3 or more times? (yes/no) | | 2/19 | 14/7 | <0.01 |
|  | F6 | Has there ever been a period of a month or more when a great deal of your time was spent using methamphetamine, getting methamphetamine, or getting over its effects? (yes/no) | | 0/21 | 12/9 | <0.01 |
| 6. Craving | F5 | Have you ever had such a strong desire for methamphetamine that it was hard to think of anything else? (yes/no) | | 0/21 | 8/13 | <0.01 |
|  | F5B | Have you ever had a strong desire or craving for methamphetamine? (yes/no) | | 0/21 | 13/8 | <0.01 |
| 7. Neuropsychiatric symptoms during MA use | F7.1 | Have you ever had a paranoid experience? (yes/no) | | 0/21 | 5/16 | <0.05 |
|  | F7.1A | Have you ever had a paranoid experience when you were using methamphetamine? (yes/no) | | 0/21 | 5/16 | <0.05 |
|  | F7.1B | Have you ever had a paranoid experience when you were not using methamphetamine? (yes/no) | | 0/21 | 0/21 | / |
|  | F8.1 | Because of your methamphetamine use, did you ever experience any of the following (yes/no): | 1. Feeling depressed or uninterested in things for more than 24 hours to the point that it interfered with your functioning? | 0/21 | 5/16 | <0.05 |
|  | F8.2 |  | 2. Having trouble concentrating or having such trouble thinking clearly for more than 24 hours that it interfered with your functioning? | 0/21 | 4/17 | 0.11 |
|  | F8.3 |  | 3. Feeling paranoid or suspicious of people for more than 24 hours to the point that it interfered with your relationships? | 0/21 | 2/19 | 0.49 |
|  | F8.4 |  | 4. Hearing, seeing, feeling, or smelling things that weren't really there? | 0/21 | 1/20 | 1 |
|  | F8.5 |  | 5. Feeling jumpy or easily startled or nervous for more than 24 hours to the point that it interfered with your functioning | 0/21 | 3/18 | 0.23 |
|  | F8A.1 | F8A Did you continue to use methamphetamine after you knew it caused this? (yes/no) | 1. Feeling depressed or uninterested in things for more than 24 hours to the point that it interfered with your functioning? | 0/21 | 3/18 | 0.23 |
|  | F8A.2 |  | 2. Having trouble concentrating or having such trouble thinking clearly for more than 24 hours that it interfered with your functioning? | 0/21 | 3/18 | 0.23 |
|  | F8A.3 |  | 3. Feeling paranoid or suspicious of people for more than 24 hours to the point that it interfered with your relationships? | 0/21 | 2/19 | 0.49 |
|  | F8A.4 |  | 4. Hearing, seeing, feeling, or smelling things that weren't really there? | 0/21 | 1/20 | 1 |
|  | F8A.5 |  | 5. Feeling jumpy or easily startled or nervous for more than 24 hours to the point that it interfered with your functioning | 0/21 | 2/19 | 0.49 |
| 8. Social | F8.6 | Decreased contact with friends or family because of your methamphetamine use? (yes/no) | | 1/20 | 12/9 | <0.01 |
|  | F8A.6 | Did you continue to use methamphetamine, though contact with friends or family members had decreased? (yes/no) | | 0/21 | 10/11 | <0.01 |
|  | F8B | Did you have decreased contact with friends or family 3 or more times in any 12-month period? (yes/no) | | 0/21 | 12/9 | <0.01 |
|  | F14A | Were there ever objections from, or problems with your family, friends, doctor, clergy, boss, or people at work or school because of your methamphetamine use? (yes/no) | | 11/10 | 18/3 | 0.02 |
| 9. Quit | F9 | Have you often wanted to stop or cut down on methamphetamine? (yes/no) | | 11/10 | 15/6 | 0.20 |
|  | F9A | Have you ever tried to stop or cut down on methamphetamine but found you couldn't? IF NEVER TRIED TO STOP/CUT DOWN, CODE NO. (yes/no) | | 0/21 | 11/10 | <0.01 |
|  | F9B | Were you unable to stop or cut down 3 or more times? (yes/no) | | 0/21 | 10/11 | <0.01 |
|  | F23 | Since the age of (ONS), has there ever been a period of time lasting 3 months or longer when you did not use methamphetamine at all? (yes/no) | | 1/20 | 14/7 | <0.01 |
| 10. Intend | F10 | Have you often used methamphetamine on more days or in larger amounts than you intended to? (yes/no) | | 0/21 | 12/9 | <0.01 |
| 11. Tolerance | F11 | Did you (a) ever need larger amounts of methamphetamine to get an effect, or did you (b) ever find that you could no longer get high on the amount you used to use? (yes/no) | | 0/21 | 10/11 | <0.01 |
| 12. Withdrawal | F12.1 | When you stopped, cut down, or went without methamphetamine, did you ever experience any of these following problems for most of the day for 2 days or longer? (yes/no) | 1. feel depressed? | 0/21 | 5/16 | <0.05 |
|  | F12.2 |  | 2. feel restless? | 1/20 | 8/13 | 0.02 |
|  | F12.3 |  | 3. feel tired, sleepy or weak? | 3/18 | 17/4 | <0.01 |
|  | F12.4 |  | 4. have trouble sleeping? | 1/20 | 5/16 | 0.18 |
|  | F12.5 |  | 5. sleep too much? | 3/18 | 17/4 | <0.01 |
|  | F12.6 |  | 6. have a strong desire or craving for methamphetamine? | 1/20 | 11/10 | <0.01 |
|  | F12.7 |  | 7. feel slowed down, like you could hardly move? | 1/20 | 5/16 | 0.18 |
|  | F12.8 |  | 8. have an increase in appetite? | 1/20 | 14/7 | <0.01 |
|  | F12.9 |  | 9. have nightmares? | 0/21 | 4/17 | 0.11 |
|  | F12A | Have you ever used methamphetamine to keep from having any these problems (or to make them go away)? (yes/no) | | 0/21 | 8/13 | <0.01 |
|  | F12B | Did this happen 3 or more times? (yes/no) | | 0/21 | 7/14 | <0.01 |
|  | F12C | Did these problems ever occur together? (yes/no) | | 3/18 | 17/4 | <0.01 |
|  | F12G | Did these problems interfere with your functioning at work, school, or home? (yes/no) | | 1/20 | 13/8 | <0.01 |
| 13. Hazard | F13 | Have you ever been under the effects of methamphetamine when it increased your chances of getting hurt, for instance, when driving a car or boat, using knives, machinery or guns, crossing against traffic, climbing or swimming? (yes/no) | | 2/19 | 12/9 | <0.01 |
|  | F13A | Have you been in situations like this 3 or more times? (yes/no) | | 1/20 | 11/10 | <0.01 |
|  | F13A.1 | Did this happen 3 or more times in any 12-month period? (yes/no) | | 1/20 | 11/10 | <0.01 |
|  | F13B | Did methamphetamine ever cause you to have any accidental injuries like a bad fall, cutting or burning yourself, or being hurt in a traffic accident? (yes/no) | | 0/21 | 1/21 | 1 |
|  | F13C | Did this happen 3 or more times? (yes/no) | | 0/21 | 0/21 | / |
|  | F13C.1 | Did this happen 3 or more times in any 12-month period? (yes/no) | | 0/21 | 0/21 | / |
| 14. Aggressive | F14B | Did you ever get into physical fights while using methamphetamine? (yes/no) | | 1/20 | 2/19 | 1 |
|  | F14C | Did (this/either of these experiences) happen 3 or more times in any 12-month period? (yes/no) | | 7/14 | 19/2 | <0.01 |
|  | F14D | Did you continue to use methamphetamine after you realized it was causing these problems? (yes/no) | | 4/17 | 19/2 | <0.01 |
| 15. Legal issues arising from MA use | F15 | Have you ever been arrested or had any other trouble with the police because of your methamphetamine use? (yes/no) | | 16/5 | 19/2 | 0.41 |
|  | F15A | Did this happen 3 or more times? (yes/no) | | 13/8 | 14/7 | 0.75 |
|  | F15B | Did this happen 3 or more times in any 12-month period? (yes/no) | | 2/19 | 3/18 | 0.63 |
| 16. Neglect of responsibilities | F16 | Has your being high on methamphetamine or experiencing its after-effects often interfered with your work, school, household, or child care responsibilities? (yes/no) | | 0/21 | 12/9 | <0.01 |
|  | F16A | Did this happen 3 or more times in any 12-month period? (yes/no) | | 0/21 | 12/9 | <0.01 |
| 17. Activities | F17 | Have you given up or greatly reduced important activities like sports, work, or associating with friends or relatives while using methamphetamine? (yes/no) | | 0/21 | 12/9 | <0.01 |
|  | F17A | Has this happened 3 or more times, or did it last a month or longer? (yes/no) | | 0/21 | 12/9 | <0.01 |
| 18. Health | F18A | Did using methamphetamine cause you to have any other problems like: An overdose? (yes/no) | | 0/21 | 1/20 | 1 |
|  | F18A1 | IF YES: Did you require medical treatment afterwards? (yes/no) | | 0/21 | 0/21 | / |
|  | F18A2 | IF YES: Did this happen 3 or more times? (overdose that required treatment) (yes/no) | | 0/21 | 0/21 | / |
|  | F18B | Other serious health problems? (yes/no) | | 0/21 | 4/17 | 0.11 |
|  | F18B.1 | IF YES: Did you continue to use methamphetamine knowing it caused health problems? (yes/no) | | 0/21 | 4/17 | 0.11 |
| 19. Seeking medical help | F24 | Did you ever bring up any problems you might have had with methamphetamine with any professional? (yes/no) | | 1/20 | 0/21 | 1 |
|  | F25 | Have you ever been treated for a problem with methamphetamine? (yes/no) | | 0/21 | 0/21 | / |
|  | F25D | Did you ever attend a self-help group for your methamphetamine use? (yes/no) | | 0/21 | 0/21 | / |
